# Comparative genome-wide association study shows single-nucleotide polymorphic *loci* associated with resistance to *Meloidogyne incognita*, *Fusarium oxysporum* f. sp. *phaseoli,* and their co-infection in common bean

**DOI:** 10.1007/s00425-026-04949-5

**Published:** 2026-03-03

**Authors:** Maria Laura Urbano Nascimento, César Júnior Bueno, Carlos Eduardo Rossi, Antônio Augusto Franco Garcia, Maria Lúcia Carneiro Vieira, Luís Eduardo Aranha Camargo, Alisson Fernando Chiorato, Sérgio Augusto Morais Carbonell, Monica Rodriguez, Luciana Lasry Benchimol-Reis

**Affiliations:** 1 Centro de Pesquisa em Recursos Genéticos Vegetais, Instituto Agronômico (IAC), Campinas, SP Brazil; 2 Divisão Avançada de Pesquisa e Desenvolvimento em Sanidade Agropecuária do Instituto Biológico, Campinas, SP Brazil; 3 Departamento de Genética, Escola Superior de Agricultura “Luiz de Queiroz”, Universidade de São Paulo, Piracicaba, SP Brazil; 4 Departamento de Fitopatologia E Nematologia, Escola Superior de Agricultura “Luiz de Queiroz”, Universidade de São Paulo, Piracicaba, SP Brazil; 5 Centro de Grãos E Fibras, Instituto Agronômico (IAC), Campinas, SP Brazil; 6 Centro de Horticultura, Instituto Agronômico (IAC), Campinas, SP Brazil; 7 The Agricultural Department of the University of Sassari, Sassari, Italy

**Keywords:** Fusarium wilt, Gall nematode, Genetic resistance, Associative mapping, SNPs

## Abstract

**Main conclusion:**

The genetic architecture underlying co-infection with *Meloidogyne incognita* and *Fusarium oxysporum* f. sp. *phaseoli* differs from that observed when these pathogens are evaluated individually.

**Abstract:**

Common bean (*Phaseolus vulgaris* L.) yield is threatened by simultaneous infection with the root-knot nematode *Meloidogyne incognita* (Mi) and *Fusarium oxysporum* f. sp. *phaseoli* (Fop). Root-knot nematodes are believed to intensify the severity of Fusarium wilt in common bean, and specific genomic regions are understood to be associated with the host response, whether conferring susceptibility or resistance. To elucidate the genetic mechanisms of this interaction, phenotypic traits were evaluated in greenhouse trials, followed by associative mapping using a genome-wide association study (GWAS) approach. The plant material consisted of a core collection of 180 common bean genotypes from the Agronomic Institute (IAC, Campinas, Brazil) diversity panel. The effects of Fop and Mi were evaluated individually and in co-infection. Associative mapping was performed using the Bayesian information and linkage disequilibrium iteratively nested keyway (BLINK) model. When plants were infected with Mi, significant SNPs were detected on chromosomes Pv07, Pv08, and Pv10 based on gall counts. SNPs were detected on Pv05, Pv06, P10, and Pv11 in association with co-infection. Regions associated with egg mass count were detected on Pv02, Pv04, and Pv05. However, co-infection revealed SNPs on chromosomes P10 and Pv11. Three SNPs were associated with Fusarium wilt–two on Pv07 and one on Pv08. The genomic regions and markers associated with resistance to Mi and Fop provide new resources for advancing understanding of host–pathogen relationships in these important pathosystems.

## Introduction

Common bean (*Phaseolus vulgaris* L.) is one of the most important legume crops worldwide, playing a crucial role in the food security of millions of people. However, its yield is often limited by soilborne pathogens, such as the root-gall nematode *Meloidogyne incognita* (Mi) and the fungus *Fusarium oxysporum* f. sp. *phaseoli* (Fop). In the field, co-infection by Fop and Mi is a considerable problem, as it causes accentuated damage to vegetative development and grain production (Kumar et al. 2017; Hua et al. 2019). Although chemical control is the most widely used method, it is not always effective. Nematicides, such as soil fumigants, are still the primary method for controlling plant parasitic nematodes (Desaeger et al. 2020), but their widespread use is increasingly being restricted, due to potential harm to animals and the environment (Lilley et al. 2011). The use of resistant cultivars represents a sustainable alternative for integrated disease management. In a molecular breeding strategy, the first step is to identify genomic *loci* involved in partial or complete resistance responses.

Genome-wide association studies (GWAS) have become a powerful approach for dissecting the genetic basis of disease resistance in plants, enabling the identification of *loci* and candidate genes associated with complex defense traits. By exploiting natural allelic variation, GWAS has successfully revealed genomic regions controlling resistance to diverse pathogens across major crops, including Fusarium crown rot in wheat (Yang et al. 2019), Fusarium rot in maize (Bian et al. 2020), and bacterial and fungal diseases in common bean (Monteiro et al. 2021; Ferreira et al. 2022). Similar advances have been achieved in rice and soybean, identifying key *loci* conferring resistance to bacterial leaf streak and *Fusarium oxysporum*, respectively (Zhu et al. 2023; Sang et al. 2023). Collectively, these studies demonstrate the versatility of GWAS as a genomic tool for identifying resistance *loci* and accelerating the development of disease-resistant cultivars through marker-assisted and genomic selection.

Resistance to Mi and Fop in common bean is generally controlled by a few genes of quantitative inheritance and modulated by complex mechanisms, including pathogen recognition, signal transduction via protein kinases, and activation of defense genes such as NBS-LRRs, chitinases, and other pathogenesis-related proteins (PRs) (Santini et al. 2016; Chen et al. 2019; Zhou and Zhang 2020; Orsi et al. 2025). In addition, the interaction between hormonal signaling pathways—notably those of salicylic acid, jasmonate, and ethylene—and post-transcriptional regulation plays a critical role in a coordinated response to biotic stresses (Chen et al. 2014; Sun et al. 2018; Thilakarathne et al. 2025). GWAS studies have revealed genomic regions in common bean individually associated with resistance to Mi, especially on chromosomes Pv01, Pv02, Pv05, Pv06, Pv07, Pv08, Pv10, and Pv11 (Giordani et al. 2021), and with resistance to Fop on Pv01, Pv03, Pv04, Pv05, Pv07, Pv08, Pv10, and Pv11 (Leitão et al. 2020; Paulino et al. 2021). These regions are often close to candidate genes involved in biotic stress responses. However, few studies have simultaneously addressed the response to multiple interactions using this approach.

The integration of precise phenotypic data and genomic variations can accelerate identification of QTLs with pleiotropic effects and facilitate gene pyramiding through marker-assisted selection. Thus, the use of GWAS on bean diversity panels offers a promising way to elucidate the genetic mechanisms underlying combined resistance to nematodes and vascular fungi, promoting significant advances in breeding for multiple tolerance to soilborne pathogens. The aim of this study was to detect *loci* and candidate genes associated with common bean defense responses against the important soilborne pathogens *Fusarium oxysporum* f. sp *phaseoli* and the root-knot nematode *Meloidogyne incognita* and discuss how these defense responses converge in co-infection.

## Materials and methods

### Plant material

The plant material consisted of a core collection of 180 genotypes from the previously described IAC diversity panel (Perseguini et al. 2016; Diniz et al. 2019). The collection includes commercial cultivars, landraces, parental lines, and populations from the crosses 'Bat 93' × 'Jalo EEP 558', 'Carioca' × 'Flor de Mayo', and 'CAL 143' × 'IAC UNA', as well as 14 F10 recombinant inbred lines (RILs) derived from the 'CAL 143' × 'IAC UNA' cross.

A total of 87 common bean lines were used from the IAC breeding program, 62 lines from the International Center for Tropical Agriculture (CIAT), 12 from the Brazilian Agricultural Research Corporation (Embrapa), 9 local Brazilian varieties, and 10 commercial cultivars. Of these, 27 genotypes are classified as being of Andean origin and 153 as of Mesoamerican origin.

### Genotypic data

SNP (single-nucleotide polymorphism) markers were selected from the genomic data obtained through genotyping by sequencing (GBS) at the Genomic Diversity Facility of the Cornell University Biotechnology Institute (USA), as described by Diniz et al. (2019).

Briefly, genomic DNA was extracted from young leaves using the CTAB protocol (CYMMIT 2005), digested with *Ape*KI, and ligated to barcoded adapters, which were pooled and amplified by PCR, as described by Elshire et al. (2011). Single-end sequencing was performed using the HiSeq 2500 platform (Illumina, San Diego, CA, USA). Sequence reads with ~ 100 bp from two DNA libraries are available in the GenBank database under BioSample SAMN05513252 and SAMN05513251, both included in BioProject PRJNA336556.

SNPs were identified and selected using the TASSEL software (Bradbury et al. 2007). The established criteria were minor allele frequency (MAF) ≥ 0.05, a minimum inbreeding coefficient of 0.9, and an identification rate < 0.9. Heterozygous *loci* were treated as missing data. A total of 10,362 SNPs were identified.

### Phenotypic data

Phenotypic evaluation of the nematode × Fop × common bean interaction was carried out in a randomized block design with three blocks, where each block constituted a replicate, and four treatments: (i) inoculation with Fop; (ii) inoculation with *Mi*; (iii) co-infection with *Mi* and Fop, and (iv) control (mock inoculation of plants with sterile distilled water, SDW). The monosporic identified culture of *Fusarium oxysporum* f. sp. *phaseoli* (Fop UFV01 isolate) was obtained from the 'Meia Noite' cultivar in Coimbra, MG, Brazil (Pereira et al. 2013; Paulino et al. 2022). The population of *Meloidogyne incognita* race 3 received from Giordani et al. (2021) was originally collected from a cotton field (*Gossypium hirsutum* L.) in the Central-West region of Brazil (15°33′6″ S, 55°10′0″ W) and subsequently multiplied from a single egg mass in the roots of a susceptible tomato line (*Solanum lycopersicum* L. ‘Santa Clara VF5600’, Sakata Seed Sudamerica®). The purity of the isolate was confirmed consistently with race 3 characteristics of reproduction in cotton, but not in tobacco (Taylor and Sasser 1978).

To prepare the seedlings, the seeds of the 180 accessions were disinfected by immersion in a 2% sodium hypochlorite solution for 2 min, followed by immersion in sterile distilled water (SDW). The seeds were sown in cell trays containing a commercial substrate (Biomix) that had been previously autoclaved twice (1 kgf/cm^2^ for 1 h each time). Ten-day-old seedlings were transplanted into 0.8 L pots containing an autoclaved mixture of soil, sand, and substrate (3:1:1).

Nematode inoculation was performed in April/2023. To prepare the inoculum, the nematodes were multiplied on susceptible tomato in a greenhouse for 60 days. Eggs were extracted by washing, cutting, and shaking the infected tomato roots for 2 min in 500 mL of 2% NaOCl solution. The suspension obtained was poured through a set of 80-, 400-, and 500-μm sieves, where the eggs were retained. The inoculum concentration was adjusted to 5000 eggs/mL by counting only the eggs on a Peters slide under an optical microscope after dilution with distilled water. For the single (Mi) and co-infection (Mi + Fop) assay, 1 mL of Mi suspension was pipetted into two 2-cm holes in the soil in the direction of the seedling root system.

To prepare the Fop inoculum, the fungus was multiplied in Petri dishes containing PDA (potato dextrose agar) culture medium. The plates were kept in a BOD chamber for 14 days at 26 °C under a 12-h photoperiod. The suspension was prepared by adding SDW to each plate, scraping the mycelium with a glass slide, and filtering the material through a double layer of gauze. The concentration was adjusted to 1 × 10^6^ conidia/mL, according to Pastor-Corrales and Abawi (1987). For the co-infection assay (Mi + Fop), Fop inoculation was performed 1 week after Mi inoculation by pipetting 1 mL of the suspension into two 2-cm holes in the soil in the direction of the seedling root system. For the Fop-only assay, plants were inoculated using the “root dip” method: before transplanting, the roots of 10-day-old seedlings were cut to about 1/3 of their length and then dipped in the inoculum suspension for 5 min (Pastor-Corrales and Abawi 1987).

The experiment was evaluated 45 days after nematode inoculation. For the Fop assay, plants were evaluated for disease severity rating (DSR) using a grading scale adapted from Pastor-Corrales and Abawi (1987) as follows: 1—no symptoms and discoloration on the hypocotyl, 3—slight discoloration on the hypocotyl, 5—intermediate discoloration on the hypocotyl, 7—severe discoloration and necrosis on the hypocotyl, and 9—plant death. Host suitability was classified as resistant when DSR = 1–3; intermediate when DSR = 3–6; and susceptible when DSR = 6–9 (Schoonhoven and Pastor-Corrales 1987).

For nematode assessment, the roots were washed, immersed in a 15 mg.L^−1^ solution of Floxin B for 15 min, and rinsed under running water. The numbers of galls and egg masses were counted per total root system under a stereoscopic microscope (10×). The degree of susceptibility of each genotype was assessed according to the 0–5 gall/eggs index scale proposed by Taylor and Sasser (1978), in which 0 = no galls or egg masses; 1 = 1–2 galls or egg masses; 2 = 3–10 galls or egg masses; 3 = 11–30 galls or egg masses; 4 = 31–100 galls or egg masses; and 5 = more than 100 galls or egg masses. Based on the gall index (GI) and egg mass index (EMI), host suitability was classified as follows: GI/EMI = 0, highly resistant; GI/EMI = 1, resistant; GI/EMI = 2, moderately resistant; GI/EMI = 3, moderately susceptible; GI/EMI = 4, susceptible; and GI/EMI = 5, highly susceptible.

The mean value of each trait per individual was calculated using the JMP®, version 14.3 (SAS Institute Inc. 2018), according to the following model:

1 $$y = Zg +Xr + e,$$

where *y* is the data vector, *g* is the vector of genotypic effects (assumed to be random), *r* is the vector of block effects (assumed to be fixed) added to the overall mean, and *e* is the vector of errors or residues (random).

The variance components were obtained using the REML (restricted maximum likelihood) procedure, and heritability was calculated via BLUPs (best linear unbiased predictors). In addition, the genotypic coefficient of variation (CVg) was calculated using the equation CVg = στ/*x̅*, where στ is the square root of the estimated genetic variance, and *x̅* is the mean value of the trait evaluated.

### Genome-wide association studies

To identify SNPs associated with the egg mass count, gall count, and Fusarium wilt severity traits, GWAS was conducted according to the Bayesian information and linkage disequilibrium iteratively nested keyway (BLINK—Huang et al. 2019) model implemented in the GAPIT package in the R software (Lipka et al. 2012).

The BLINK method uses two fixed-effect models (FEMs) and a filtering process that selects a set of pseudo-QTNs (quantitative trait nucleotides) that are not in LD (linkage disequilibrium) with each other, which are used as covariates. The entire sequence is iterated until all genetic markers have been tested and the selection of pseudo-QTNs is optimized. The first FEM tests M genetic markers, one at a time. The pseudo-QTNs are included as covariates to simultaneously control false positives and reduce false negatives. Specifically, the first FEM can be expressed as follows:

2 $$yi = S\times i1b1 + S\times i2b2 + ... + S\times ikbk + Sijdj + ei,$$

where yi is the observation of the i-th individual; Si1, Si2,..., Sik are the genotypes of k pseudo-QTNs, beginning as an empty set; b1, b2,..., bk are the corresponding effects of the pseudo-QTNs; Sij is the genotype of the i-th individual and the j-th genetic marker; dj is the effect of the j-th genetic marker; and ei is the residual, with a distribution of mean zero and variance of σ2e.

Associations were tested for the 10,362 SNP markers, and the adjusted means of each trait were obtained according to the phenotypic model described above, but treating the genotype as a fixed effect to obtain the best linear unbiased estimators (BLUEs) to avoid double shrinkage. The significance threshold for detecting SNPs was determined using Bonferroni's multiple test correction (*p* < 4.82e-06) and permutation tests with 1,000 interactions, both with 5% global significance for Type I error.

### GO enrichment analysis and search for candidate genes

The reference genome of *P. vulgaris* version 2.1 (Schmutz et al. 2014), available on the Phytozome platform, was used to search for candidate genes potentially involved in the responses studied. The intervals of genomic *loci* in LD with the significant SNPs identified through the GWAS results were considered. The LD for this population (*r*^2^ = 1 Mbp) was previously calculated and described by Diniz et al. (2019).

To infer candidate genes, we compared the set of genes found in genomic *loci* identified by GWAS in this study with those described as differentially expressed at 4 and 10 days post-inoculation (DPI) by Santini et al. (2016) in response to Mi infection, and at 24 h post-inoculation (HPI) by Chen et al. (2019) in response to Fop infection.

## Results

### Phenotypic analyses

At 45 days after nematode inoculation, genotypes with more resistant or more susceptible reactions could be distinguished. From 175 accessions suitable for GWAS, 113 were resistant and 62 intermediate or susceptible to Fop when pathogen was inoculated alone, and 129 were resistant and 46 susceptible or intermediate in co-infection with Mi considering the DSR. Regarding galls and egg masses indexes, respectively, 93 and 43 accessions were found to be resistant, and 82 and 132 highly, moderately, or susceptible when nematode was inoculated alone. In co-infection with Fop, 74 and 43 were resistant, while 101 and 132 highly, moderately, or susceptible for galls and egg mass indexes, respectively (Table 1).

**Table 1:** Host suitability of 175 bean genotypes used in genome-wide association studies for resistance to *Meloigogyne incognita* (Mi) and/or *Fusarium oxysporum* f. sp. *phaseoli* (Fop)

Germplasm	Fusarium wilt severity				Galls count				Egg mass count			
	DSR		Reaction class^a^		GI		Reaction class^b^		EMI		Reaction class^b^	
	Single	Double	Single	Double	Single	Double	Single	Double	Single	Double	Single	Double
Frijol_Negro	2.0	2.3	R	R	3	3	MS	MS	4	4	S	S
Feijao_Suico	2.3	1.0	R	R	1	2	R	MR	3	3	MS	MS
Chileno_Branco	1.0	2.3	R	R	4	4	S	S	4	4	S	S
ECU_311	3.7	1.7	I	R	4	4	S	S	4	4	S	S
Vermelhinho	1.0	4.3	R	I	4	4	S	S	4	4	S	S
Bagajo	1.7	3.0	R	I	4	4	S	S	4	3	S	MS
Mexico_155	3.7	1.0	I	R	3	4	MS	S	4	4	S	S
Baetao_30273	5.0	1.0	I	R	3	3	MS	MS	4	4	S	S
Preto_208	1.0	3.7	R	I	3	1	MS	R	3	2	MS	MR
Preto_184	1.7	1.0	R	R	2	1	MR	R	2	2	MR	MR
Honduras_32	3.0	1.0	I	R	3	4	MS	S	3	4	MS	S
Guatemala_479	2.3	1.7	R	R	2	2	MR	MR	3	3	MS	MS
Jamapa_CNF_1671	8.0	1.7	S	R	4	3	S	MS	4	3	S	MS
Mulatinho_VP_102	4.3	1.0	I	R	3	3	MS	MS	4	4	S	S
Tupi	7.0	1.7	S	R	3	3	MS	MS	3	4	MS	S
Rosinha_G2	4.0	1.0	I	R	1	2	R	MR	3	3	MS	MS
Preto_do_Procone	2.3	1.0	R	R	2	3	MR	MS	3	4	MS	S
Porrillo_1	5.0	1.0	I	R	3	3	MS	MS	4	3	S	MS
Mexico_498	2.3	3.0	R	I	0	2	HR	MR	2	3	MR	MS
Small_White_59_Preto	1.7	1.7	R	R	2	3	MR	MS	3	4	MS	S
Perry Marron	1.7	1.7	R	R	1	3	R	MS	3	4	MS	S
Mortino	2.3	1.0	R	R	1	2	R	MR	3	3	MS	MS
Rosado_13	3.0	1.0	I	R	0	2	HR	MR	1	3	R	MS
Porrillo_Sintetico	1.0	1.0	R	R	2	3	MR	MS	3	3	MS	MS
Puebla_152_CIAT	3.0	2.3	I	R	0	0	HR	HR	2	0	MR	HR
Jalo_110	5.7	3.7	I	I	2	2	MR	MR	3	4	MS	S
IAC_Maravilha	2.3	1.0	R	R	3	3	MS	MS	4	4	S	S
FEB_179	5.0	1.7	I	R	3	4	MS	S	4	4	S	S
Jamapa_CIAT	5.0	1.0	I	R	1	1	R	R	3	2	MS	MR
Puebla_152_CNF_1807	2.3	1.0	R	R	1	0	R	HR	2	2	MR	MR
EMP_81	1.7	3.7	R	I	3	3	MS	MS	4	4	S	S
Jalo_110	1.0	3.7	R	I	4	3	S	MS	4	3	S	MS
ARC_3	1.0	1.0	R	R	2	3	MR	MS	3	3	MS	MS
ARC_4	1.7	1.0	R	R	2	2	MR	MR	3	3	MS	MS
LP_90_91_R_Bac	1.0	1.0	R	R	3	3	MS	MS	3	4	MS	S
EMP_407	6.0	1.0	S	R	3	3	MS	MS	4	4	S	S
FEB_180	3.0	5.0	I	I	1	2	R	MR	2	1	MR	R
Alemao	2.3	4.3	R	I	0	2	HR	MR	2	2	MR	MR
Pinto_114	3.0	1.0	I	R	2	3	MR	MS	3	3	MS	MS
Flor_de_Mayo	3.0	1.0	I	R	2	2	MR	MR	2	2	MR	MR
G2333	4.3	2.0	I	R	3	2	MS	MR	3	2	MS	MR
DOR_390	2.3	1.0	R	R	1	2	R	MR	2	3	MR	MS
DOR_476	2.3	4.3	R	I	2	2	MR	MR	2	3	MR	MS
Turrialba_1	6.0	5.0	S	I	1	2	R	MR	3	3	MS	MS
AND_279	3.0	5.0	I	I	1	3	R	MS	2	3	MR	MS
RAZ_49	2.3	6.3	R	S	1	0	R	HR	2	2	MR	MR
RAZ_55	1.7	3.7	R	I	0	0	HR	HR	0	1	HR	R
Batista_Brilhante_CB	3.0	1.0	I	R	0	1	HR	R	0	3	HR	MS
A_449	1.7	5.7	R	I	3	4	MS	S	3	4	MS	S
Apore	3.0	3.0	I	I	1	3	R	MS	2	4	MR	S
Branquinho	5.0	1.0	I	R	3	3	MS	MS	3	3	MS	MS
BRS_Cometa	3.0	1.0	I	R	3	4	MS	S	4	4	S	S
BRS_Horizonte	3.7	5.7	I	I	3	4	MS	S	3	4	MS	S
BRS_Pontal	3.0	1.0	I	R	4	4	S	S	3	4	MS	S
BRSMG_Talisma	3.0	2.3	I	R	3	3	MS	MS	3	4	MS	S
Campeao_II	3.7	5.7	I	I	0	2	HR	MR	3	3	MS	MS
Caneludo	1.7	1.0	R	R	3	3	MS	MS	4	4	S	S
Carioca_Comum	1.7	1.7	R	R	1	2	R	MR	2	3	MR	MS
Carioca_Lustroso	3.0	1.0	I	R	3	3	MS	MS	4	4	S	S
Carioca_MG	1.0	2.3	R	R	2	2	MR	MR	3	3	MS	MS
Carioca_Precoce	1.0	1.7	R	R	2	2	MR	MR	2	4	MR	S
H96A28_P4_1_1_1_1	2.3	2.3	R	R	3	4	MS	S	4	4	S	S
H96A102_1_1_152	2.3	1.0	R	R	1	2	R	MR	3	3	MS	MS
H96A31_P2_1_1_1_1	1.0	3.0	R	I	3	3	MS	MS	3	4	MS	S
IAC_Alvorada	1.7	1.0	R	R	1	3	R	MS	3	3	MS	MS
IAC_Apua	1.0	2.3	R	R	1	3	R	MS	3	3	MS	MS
IAC_Ayso	3.0	1.7	I	R	3	2	MS	MR	3	4	MS	S
IAC_Carioca	2.3	2.3	R	R	3	3	MS	MS	2	3	MR	MS
IAC_Carioca_Akyta	2.0	1.7	R	R	2	4	MR	S	3	4	MS	S
IAC_Carioca_Arua	1.7	1.0	R	R	1	2	R	MR	3	3	MS	MS
IAC_Carioca_Pyata	3.7	1.7	I	R	0	1	HR	R	2	2	MR	MR
IAC_Carioca_Tybata	1.0	1.0	R	R	0	1	HR	R	1	2	R	MR
IAC_Votuporanga	1.7	1.0	R	R	2	2	MR	MR	3	1	MS	R
IAC_Ybata	1.7	1.7	R	R	3	3	MS	MS	3	4	MS	S
IAPAR_81	2.3	1.0	R	R	1	3	R	MS	2	3	MR	MS
IAPAR_31	3.0	3.7	I	I	4	4	S	S	4	4	S	S
Perola	1.0	1.7	R	R	2	2	MR	MR	2	3	MR	MS
T0	1.0	2.3	R	R	4	3	S	MS	4	4	S	S
Gen05P3_1_6_1	1.7	6.0	R	S	2	4	MR	S	3	4	MS	S
Gen05P4_2_6_2	4.3	3.7	I	I	0	2	HR	MR	1	3	R	MS
Gen05P5_3_8_1	2.3	3.7	R	I	2	2	MR	MR	3	3	MS	MS
Gen05P5_3_8_2	2.3	3.0	R	I	2	1	MR	R	3	2	MS	MR
Gen05P5_4_8_2	1.0	2.3	R	R	1	3	R	MS	2	3	MR	MS
Gen05Pr11_2_3_1	3.7	7.0	I	S	2	1	MR	R	3	0	MS	HR
Gen05Pr11_2_13_1	1.7	3.7	R	I	2	2	MR	MR	2	3	MR	MS
Gen05Pr11_2_14_2	1.7	6.3	R	S	3	2	MS	MR	4	2	S	MR
Gen05Pr11_3_5_1	1.0	1.7	R	R	0	2	HR	MR	3	2	MS	MR
Branco_Argentino	3.0	5.0	I	I	3	2	MS	MR	3	2	MS	MR
Gen05PR13_1_8_1_2	3.0	2.3	I	R	2	2	MR	MR	3	2	MS	MR
Gen05PR13_1_8_1_1	1.0	1.7	R	R	3	2	MS	MR	3	3	MS	MS
Gen05PR13_1_6_1_2	1.0	4.3	R	I	2	1	MR	R	3	2	MS	MR
Gen05PR13_2_2_1_2	2.3	3.0	R	I	1	0	R	HR	2	0	MR	HR
Gen05C1_3_2_1_1	1.0	2.3	R	R	1	2	R	MR	1	3	R	MS
Gen05C1_3_3_1_1	4.3	2.3	I	R	2	2	MR	MR	3	3	MS	MS
Gen05C2_1_6_1_1	4.3	1.0	I	R	4	3	S	MS	4	3	S	MS
Gen05C2_1_1_1_3	1.0	3.7	R	I	0	3	HR	MS	0	3	HR	MS
Gen05C2_1_1_1_1	2.3	1.7	R	R	3	3	MS	MS	4	4	S	S
Gen05C3_2_4_1_1	3.7	1.0	I	R	3	3	MS	MS	4	4	S	S
Gen05C3_2_4_1_7	1.7	1.0	R	R	2	2	MR	MR	3	3	MS	MS
Gen05C4_3_1_1_2	1.7	1.7	R	R	3	3	MS	MS	4	4	S	S
Gen05C4_3_1_1_1	3.0	1.7	I	R	1	2	R	MR	2	3	MR	MS
Gen05C4_4_3_1_2	2.3	1.7	R	R	3	4	MS	S	3	4	MS	S
Gen05C4_6_2_1_2	1.0	3.7	R	I	3	4	MS	S	3	4	MS	S
Gen05C5_1_2_2_2	1.0	2.0	R	R	3	3	MS	MS	2	3	MR	MS
Gen05C5_2_10_1_1	1.0	1.0	R	R	0	4	HR	S	0	4	HR	S
Gen05C6_3_5_2_1	3.7	1.7	I	R	0	2	HR	MR	2	2	MR	MR
Gen05C6_4_5_1_2	2.3	2.3	R	R	2	2	MR	MR	3	3	MS	MS
UxC_1_19	1.7	5.0	R	I	4	4	S	S	4	4	S	S
Gen05C7_3_2_2_2	4.3	2.3	I	R	3	3	MS	MS	4	3	S	MS
UxC_1_1	4.0	4.3	I	I	3	3	MS	MS	4	3	S	MS
UxC_2_20	3.0	1.7	I	R	2	4	MR	S	2	4	MR	S
UxC_1_2	1.7	3.0	R	I	4	4	S	S	4	4	S	S
UxC_1_5	1.7	1.7	R	R	2	3	MR	MS	4	4	S	S
UxC_9_16	2.3	3.0	R	I	4	4	S	S	4	4	S	S
CxU_1_5	2.3	4.0	R	I	3	4	MS	S	4	4	S	S
CxU_1_7	1.7	2.3	R	R	3	4	MS	S	3	4	MS	S
CxU_1_19	1.0	2.0	R	R	3	4	MS	S	4	4	S	S
CxU_2_11	1.0	1.0	R	R	4	4	S	S	4	4	S	S
CxU_7_8	1.7	1.0	R	R	4	3	S	MS	4	3	S	MS
UxC_2_18	2.3	3.0	R	I	4	3	S	MS	4	3	S	MS
UxC_3_3	2.3	5.0	R	I	3	4	MS	S	4	3	S	MS
VAX_1	1.0	1.0	R	R	0	2	HR	MR	2	3	MR	MS
A_0774	1.0	1.7	R	R	2	3	MR	MS	3	4	MS	S
BAT_447	3.0	1.0	I	R	3	3	MS	MS	4	3	S	MS
SEA_5	1.7	1.0	R	R	2	2	MR	MR	4	3	S	MS
IAC_Una	3.0	2.3	I	R	3	3	MS	MS	4	3	S	MS
CAL_143	1.0	1.0	R	R	3	4	MS	S	3	4	MS	S
Sanilac	4.0	1.0	I	R	3	3	MS	MS	4	4	S	S
Red_Kidney	1.7	1.0	R	R	3	4	MS	S	4	4	S	S
FEB_176	5.7	1.7	I	R	2	2	MR	MR	3	3	MS	MS
FEB_177	3.0	1.0	I	R	2	3	MR	MS	3	2	MS	MR
J_61_5_3_1	1.7	1.0	R	R	3	3	MS	MS	3	4	MS	S
J_43_5_1	2.3	1.0	R	R	2	1	MR	R	3	2	MS	MR
J_43_1_1_1	1.7	1.7	R	R	3	4	MS	S	4	4	S	S
J_39_1_3_2	2.3	1.0	R	R	2	2	MR	MR	2	3	MR	MS
E_20_2_1	1.7	3.0	R	I	2	1	MR	R	3	3	MS	MS
ARA_1	4.0	3.0	I	I	2	1	MR	R	3	1	MS	R
X29_24_6_1_1	1.0	1.0	R	R	2	3	MR	MS	3	3	MS	MS
AND_277	1.0	2.3	R	R	3	4	MS	S	4	4	S	S
Brasil_2	3.7	3.0	I	I	3	3	MS	MS	3	4	MS	S
Rio_Tibagi	2.3	1.7	R	R	3	3	MS	MS	4	3	S	MS
Great_Northern	2.3	2.3	R	R	3	3	MS	MS	4	4	S	S
Apetito_Blanco	3.0	1.7	I	R	1	3	R	MS	2	3	MR	MS
Garbancillo	2.0	1.0	R	R	4	3	S	MS	4	4	S	S
TU	1.0	1.0	R	R	3	3	MS	MS	4	4	S	S
Barbunya	1.0	1.0	R	R	3	4	MS	S	3	4	MS	S
Red_Mexican	2.3	1.0	R	R	2	4	MR	S	3	4	MS	S
Serro_Azul_Fosco	3.7	1.0	I	R	3	3	MS	MS	3	3	MS	MS
Serro_Azul_Brilhante	4.0	1.7	I	R	2	2	MR	MR	3	2	MS	MR
Jalo_Precoce	1.0	2.3	R	R	3	3	MS	MS	3	3	MS	MS
BRS_Pitanga	2.3	2.3	R	R	3	4	MS	S	4	4	S	S
BRS_Vereda	2.3	1.0	R	R	1	3	R	MS	3	3	MS	MS
Pompadour	2.3	1.0	R	R	3	4	MS	S	3	4	MS	S
Antioquia_8	1.0	1.7	R	R	2	3	MR	MS	3	3	MS	MS
Jabola	2.3	2.3	R	R	4	4	S	S	4	4	S	S
LP_09_40	1.0	3.0	R	I	2	3	MR	MS	4	3	S	MS
Gen_C_10_2_4_41	1.0	1.0	R	R	3	2	MS	MR	3	3	MS	MS
IPR_Tangara	3.7	1.0	I	R	3	4	MS	S	4	4	S	S
Gen_FAP_F3_2	1.7	1.0	R	R	3	4	MS	S	3	4	MS	S
Gen_P10_1_9_38	1.7	1.0	R	R	3	2	MS	MR	5	3	HS	MS
Gen_49_61_1_2	1.7	1.0	R	R	2	2	MR	MR	3	3	MS	MS
Gen_45_57_7_3_1_4	5.7	2.3	I	R	1	1	R	R	2	2	MR	MR
Gen_C_10_2_16_8	1.0	1.7	R	R	1	1	R	R	3	2	MS	MR
Gen_41_35_3_2_2	3.7	1.0	I	R	2	3	MR	MS	3	3	MS	MS
Gen_FAP_F3_RC2_2	1.7	1.0	R	R	1	2	R	MR	2	3	MR	MS
Gen_45_57_4_2_1_4	1.7	1.0	R	R	2	2	MR	MR	2	2	MR	MR
Gen_87_73_5_3_1	1.7	1.0	R	R	2	2	MR	MR	3	2	MS	MR
IAC_Milenio	1.0	2.3	R	R	2	2	MR	MR	2	2	MR	MR
Gen_45_57_7_3_1_2	1.0	2.3	R	R	2	1	MR	R	2	1	MR	R
CNFC_10762	1.0	1.0	R	R	0	2	HR	MR	0	2	HR	MR
LEC_01_11	3.0	1.0	I	R	1	3	R	MS	1	3	R	MS
FT_08_75	3.0	1.0	I	R	3	3	MS	MS	4	3	S	MS
CHC_98_42	1.0	5.0	R	I	1	2	R	MR	3	2	MS	MR
FT_08_47	3.7	3.7	I	I	2	1	MR	R	3	2	MS	MR
TB_01_13	3.0	3.7	I	I	3	1	MS	R	3	0	MS	HR

^a^Reaction class was based on the Schoonhoven and Pastor-Corrales (1987) DSR scale, where 1–3 = resistant (R), 3.1–6 = intermediate (I), and 6.1–9 = susceptible (S)

^b^Reaction class was based on the 0–5 gall/egg mass index scale proposed by Taylor and Sasser (1978),

where 0 = highly resistant; 1 = resistant; 2 = moderately resistant; 3 = moderately susceptible; 4 = susceptible and 5 = highly susceptible

The traits associated with nematode reproduction showed high coefficients of genetic variation and heritability, particularly for gall count (77.34% and 70.10%). However, lower coefficients were observed for the response to Fusarium wilt (39.81% and 29.06%). Heritability estimates were also lower for this trait (52.61% and 21.97%), indicating a greater influence of environmental factors on Fusarium wilt response in common bean (Table 2).

**Table 2:** Descriptive summary and variance component estimates for the count of galls, count of egg masses and severity of Fusarium wilt in a panel of bean diversity 45 days after inoculation with *M. incognita* and/or *F. oxysporum* f. sp. *phaseoli* (Fop)

				Variance component estimates		
Trait	Inoculum	Amplitude	CV_g_(%)	Genotype	Residual	H^2^_a_(%)
Count of galls	*M. incognita*	0–117	77.34	117.24	167.69	67.72
	*M. incognita* + Fop	0–110	70.10	177.39	184.65	74.24
Count of egg masses	*M. incognita*	0–188	49.46	205.72	527.33	53.92
	*M. incognita* + Fop	0–151	48.46	184.08	379.64	59.26
Fusarium wilt severity	Fop	1–9	39.81	0.91	2.47	52.61
	*M. incognita* + Fop	1–9	29.06	0.41	4.35	21.97

^1^CVg = coefficient of genetic variation; H^2^_a_ = heritability in the broad sense

### Genome-wide association studies

Based on the Bonferroni correction, significant SNPs were detected that were associated with the resistance/susceptibility responses when Mi or Fop was inoculated individually. Regarding co-infection, no markers associated with the severity of Fusarium wilt were detected. More than one genomic region was associated with the same trait, and the associations varied depending on the inoculum procedure—whether single pathogen or co-infection.

### Gall count

**Fig. 1:**
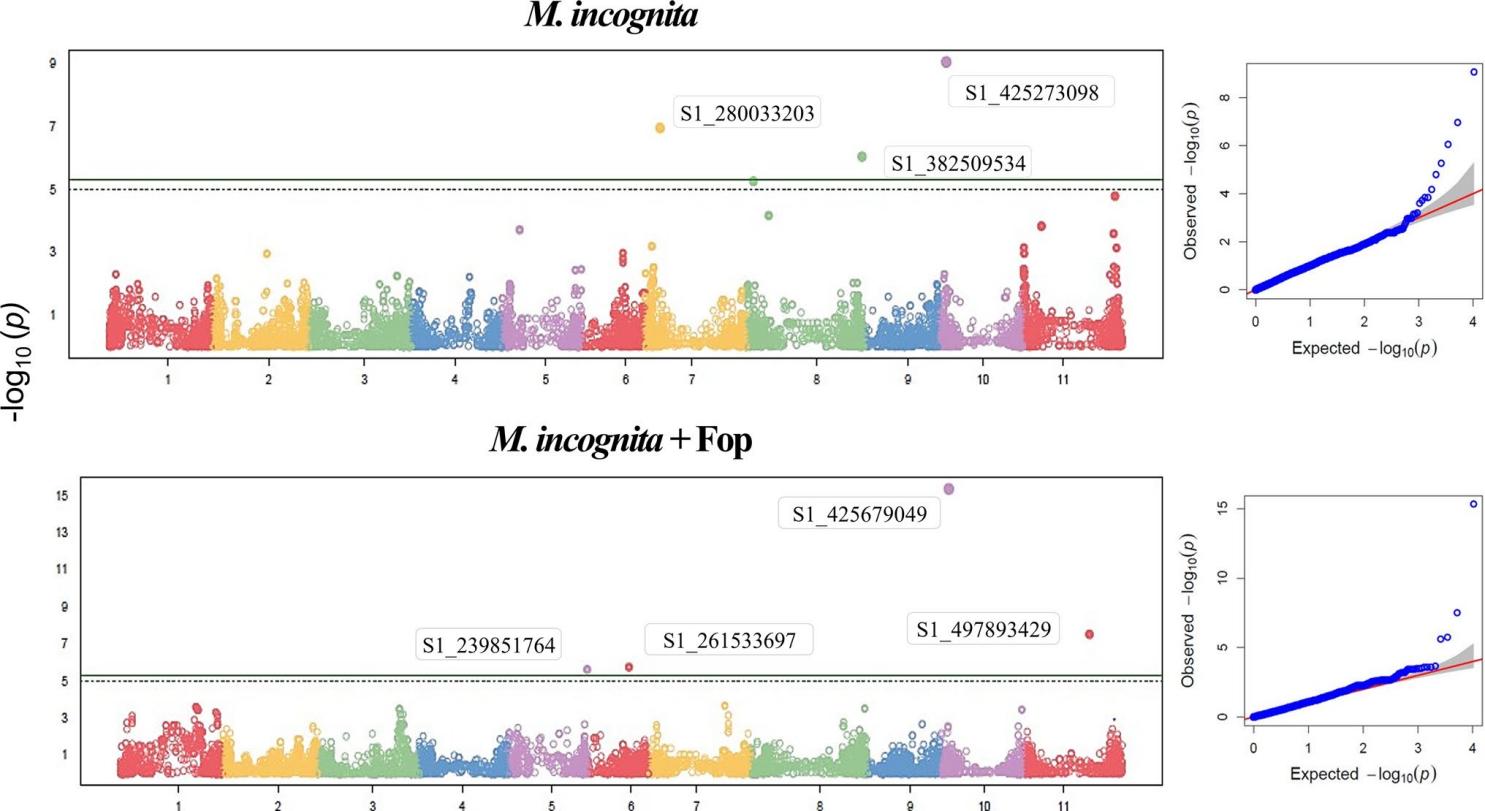
GWAS of gall counts in a common bean diversity panel 45 days after inoculation with *Meloidogyne incognita* (Mi) or co-inoculation with Mi and *Fusarium oxysporum* f. sp. *phaseoli* (Fop)

Regarding gall count, three SNPs were detected when Mi was inoculated alone on plants, and four SNPs when plants were co-infected with Mi and Fop. Additionally, when the threshold was defined based on the permutation test, one additional SNP was found when only Mi was inoculated on the plant (Figure 1).

The genomic regions identified differed, except for the region on chromosome Pv10. In plants inoculated with nematodes, SNPs were detected on chromosomes Pv07, Pv08, and Pv10. Regarding the plants with Mi + Fop, significant SNPs were identified on chromosomes Pv05, Pv06, Pv10, and Pv11 (Table 3).

**Table 3:** SNP markers associated with the count of galls in an IAC bean diversity panel 45 days after inoculation with *Meloidogyne incognita* or interaction *M. incognita* plus *Fusarium oxysporum* f. sp. *phaseoli* (Fop)

Marker name	Chr	Position (bp)	*p* value	MAF	Allelic refference	Allelic variant	Effect of variant allele	PVE (%)
*M. incognita*								
S1_280033203	Pv07	7,745,264	1.10E-07	0.10	T	G	− 7.66	29.04
S1_382509534	Pv08	58,462,973	8.95E-07	0.08	G	A	5.83	11.34
S1_425273098	Pv10	4,094,197	8.80E-10	0.29	A	C	4.93	6.40
*M. incognita* + Fop								
S1_239851764	Pv05	40,360,567	2.43E-06	0.12	T	C	6.08	7.83
S1_261533697	Pv06	21,223,114	1.78E-06	0.21	C	G	5.82	7.20
S1_425679049	Pv10	4,500,148	4.48E-16	0.48	C	T	−7.79	13.40
S1_497893429	Pv011	33,439,277	3.05E-08	0.09	G	A	8.78	16.48

^1^*Chr* chromosome, *MAF* minor allele frequency, *PVE* proportion of the variance explained by the SNP–trait association

### Egg mass count

**Fig. 2:**
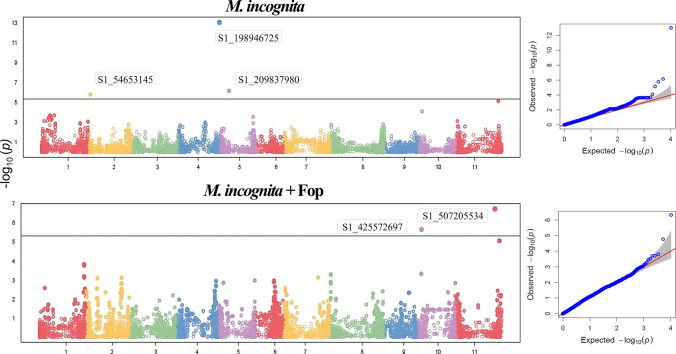
GWAS of egg mass counts in a common bean diversity panel 45 days after inoculation with *Meloidogyne incognita* (Mi) or co-inoculation with Mi and *Fusarium oxysporum* f. sp. *phaseoli* (Fop)

Regarding egg mass count, three SNPs were found when the nematode was inoculated alone, and only two when co-inoculated with Fop. No additional SNPs were detected when the threshold was set according to the permutation test (Figure 2).

Similar to the host response for gall count, the genomic regions related to egg mass count diverged depending on whether the inoculum was Mi or Mi + Fop. Significant SNPs were detected on chromosomes Pv02, Pv04, and Pv05 in the former case, and on chromosomes P10 and Pv11 in the latter (Table 4).

**Table 4:** SNP markers associated with the count of egg masses in an IAC bean diversity panel 45 days after inoculation with *Meloidogyne incognita* or interaction *M. incognita* plus *Fusarium oxysporum* f. sp. *phaseoli* (Fop)

Marker name	Chr	Position (bp)	*p* value	MAF	Allelic refference	Allelic variant	Effect of variant allele	PVE (%)
*M. incognita*								
S1_54653145	Pv02	2,447,514	1.63E-06	0.16	A	T	9.94	9.45
S1_198946725	Pv04	45,415,647	9.13E-14	0.11	T	C	− 29.34	60.16
S1_209837980	Pv05	10,346,783	6.87E-07	0.26	T	A	6.26	3.71
*M. incognita* + Fop								
S1_425572697	Pv10	4,393,796	2.16E-06	0.49	A	T	− 6.28	11.84
S1_507205534	Pv11	42,751,382	1.78E-07	0.09	G	A	12.06	34.36

^1^*Chr* chromosome, *MAF* minor allele frequency, *PVE* proportion of the variance explained by the SNP–trait association

### Fusarium wilt severity

**Fig. 3:**
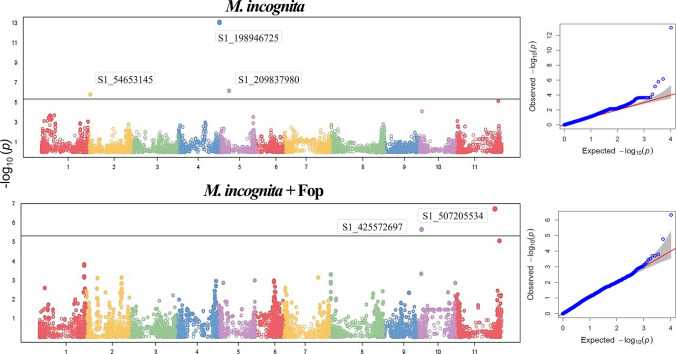
GWAS of Fusarium wilt severity in an IAC common bean diversity panel 45 days after inoculation with *Fusarium oxysporum* f. sp. *phaseoli* (Fop)

Three SNPs were detected in plants inoculated with Fop. However, in plants inoculated with Mi and Fop, no markers associated with the trait were detected (Fig. 3).

Three SNPs associated with Fusarium wilt were detected—two on chromosome Pv07 and one on Pv08. The region associated with this trait on Pv08 is near that identified for gall count on the same chromosome (Table 5).

**Table 5:** SNP markers associated with Fusarium wilt severity in an IAC bean diversity panel 45 days after inoculation *Fusarium oxysporum* f. sp. *phaseoli* (Fop)

Marker name	Chr	Position (bp)	*p* value	MAF	Allelic refference	Allelic variant	Effect of variant allele	PVE (%)
S1_282967214	Pv07	10,679,275	9.89E-07	0.44	G	T	−0.47	7.08
S1_310700629	Pv07	38,412,690	1.74E-09	0.40	C	T	−0.58	11.39
S1_381064496	Pv08	57,017,935	1.05E-06	0.11	A	G	0.60	11.65

^1^*Chr* chromosome, *MAF* minor allele frequency, *PVE* proportion of the variance explained by the SNP–trait association

### GO enrichment analysis

**Fig. 4:**
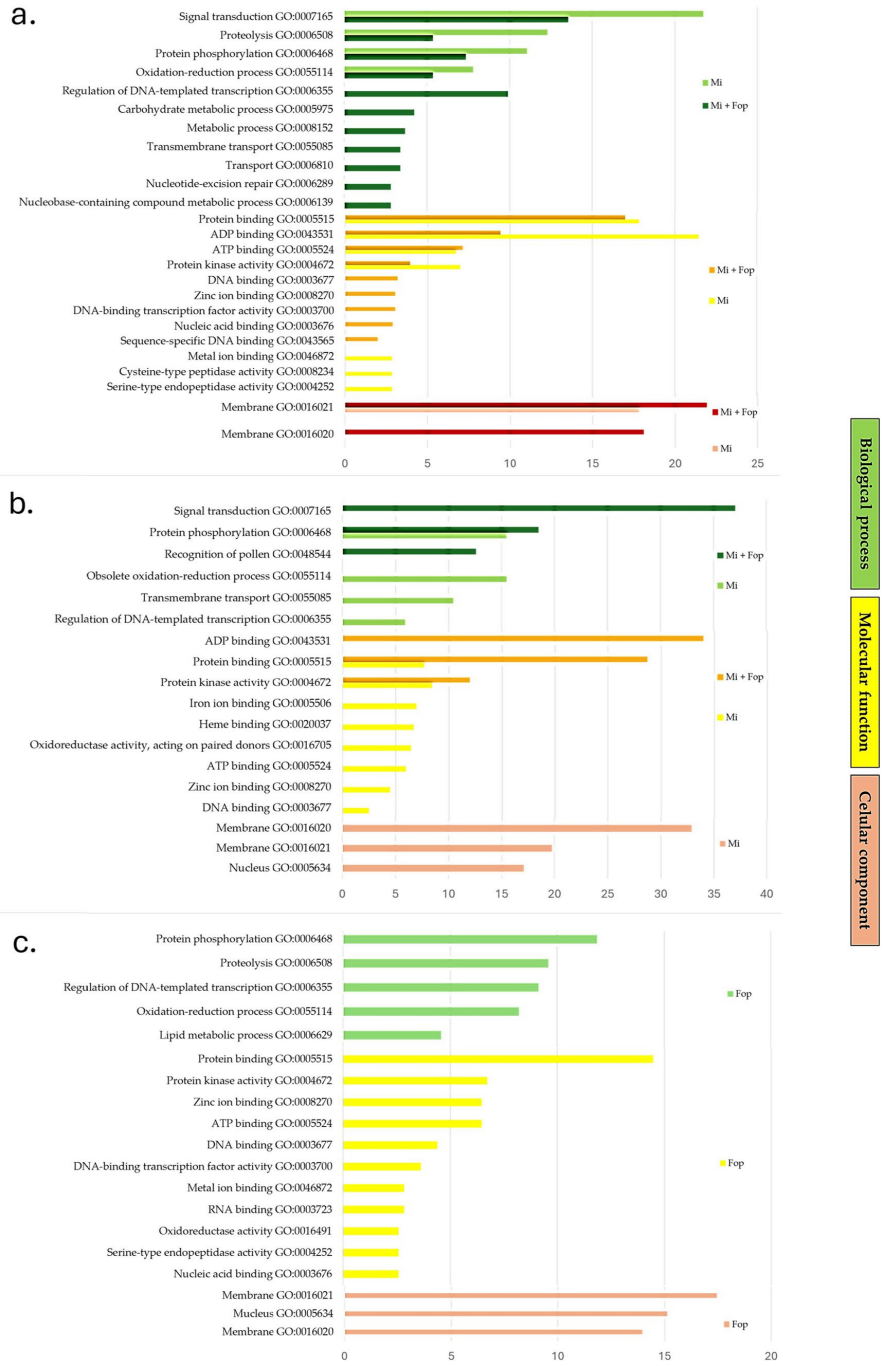
GO enrichment analysis for **a** gall count, **b** egg mass count, and **c** Fusarium wilt severity in a common bean diversity panel 45 days after inoculation with *Meloidogyne incognita* (Mi) or with *Fusarium oxysporum* f. sp. *phaseoli* (Fop) or co-inoculation with *M. incognita* and *Fusarium oxysporum* f. sp. *phaseoli* (Mi + Fop)

Genes located within 1 Mbp of the SNPs identified by GWAS were investigated using the Phytozome database (Schmutz et al. 2014). Based on gene ontology (GO) enrichment analyses, the host responses regarding gall count and egg mass count differed when plants were inoculated with Mi alone or Mi + Fop. The results of these analyses preferentially showed components involved in signal recognition, signaling, and defense pathways (Fig. 4).

In genomic regions associated with gall count, the main GO terms for biological processes were signal transduction (22% Mi and 14% Mi + Fop), proteolysis (12% Mi and 5% Mi + Fop), protein phosphorylation (11% Mi and 7% Mi + Fop), and the oxidation–reduction processes (8% Mi and 5% Mi + Fop). In plants co-infected with Mi and Fop, regulation of DNA-templated transcription (10%) was another important biological process, as well as transport functions and metabolic processes. The most enriched molecular functions for this trait were protein binding (18% Mi and 17% Mi + Fop), ADP binding (21% Mi and 9% Mi + Fop), ATP binding (7% Mi and 7% Mi + Fop), and protein kinase activity (7% Mi and 4% Mi + Fop). The most relevant cellular component was the membrane (18% Mi and 22% Mi + Fop).

For genomic regions associated with egg mass count, the most enriched biological process common to both Mi and Mi + Fop was protein phosphorylation (15% Mi and 19% Mi + Fop). For co-infection, signal transduction was the most enriched process (37%), followed by pollen recognition (13%). Transmembrane transport, oxidation–reduction processes, and regulation of DNA-templated transcription were enriched only under Mi inoculation alone. The most enriched molecular functions for egg mass count were protein binding (8% Mi and 29% Mi + Fop) and protein kinase activity (8% Mi and 12% Mi + Fop), common to both Mi and Mi + Fop. ATP binding (6% Mi) was important under Mi inoculation, whereas ADP binding was specific to Mi + Fop. The most enriched cellular components were the membrane (33%) and nucleus (17%), both identified only in plants inoculated with Mi alone.

### Search for candidate genes

Considering the genes described as differentially expressed in common bean after infection by Mi (Santini et al. 2016) and Fop (Chen et al. 2019), a total of 24 RGAs were identified across chromosomes Pv02, Pv06, Pv07, Pv08, and Pv10—4 associated with Mi, 6 with Fop, and 14 with co-infection. These candidate genes contain conserved domains known to play key roles in plant defense mechanisms and signaling pathways (Table 6).

**Table 6:** Candidate genes identified by the GWAS-associated genomic regions within the intervals in linkage disequilibrium with the SNP markers identified and differentially expressed in common bean roots 24 h post-inoculation (HPI) with *Fusarium oxysporum* f. sp. *phaseoli* (Chen et al. 2019), 4 and/or 10 days post-inoculation (DPI) with *Meloidogyne incognita* (Santini et al. 2016)

Trait	Associated marker	Gene	Functional annotation	4DPI	10DPI	24HPI
*M. incognita*						
CG	S1_382509534	Phvul.008G235100	Signaling calcium	−1.06	-	-
CEM	S1_54653145	Phvul.002G017500	Secondary metabolism phenylpropanoids.lignin biosynthesis CCoAOMT	1.88	-	-
	S1_54653145	Phvul.002G033300	Secondary metabolism.flavonoids isoflavones isoflavone reductase	1.57	-	-
Fop						
FWS	S1_282967214	Phvul.007G095000	Auxin response factor	*-*	*-*	−3.00
	S1_310700629	Phvul.007G254200	Disease resistance protein (NB-ARC domain containing)	*-*	*-*	3.06
	S1_310700629	Phvul.007G254501	Disease resistance protein (NB-ARC domain containing)	*-*	*-*	−3.20
	S1_310700629	Phvul.007G254700	Disease resistance protein (NB-ARC domain containing)	*-*	*-*	−7.57
	S1_310700629	Phvul.007G257800	Calcium-dependent lipid-binding (CaLB domain) plant phosphoribosyltransferase family protein	*-*	*-*	−8.39
	S1_310700629	Phvul.008G213200	Leucine-rich repeat receptor-like protein kinase family protein	*-*	*-*	2.95
*M. incognita* + Fop						
CG/FWS	S1_261533697	Phvul.006G094500	Redox ascorbate and glutathione glutathione	-	1.07	−2.92
CG/FWS	S1_261533697	Phvul.006G109000	Stress biotic PR-proteins	-	−3.87	−3.39
CG	S1_261533697	Phvul.006G101300	Hormone metabolism auxin induced–regulated–responsive–activated	1.00	-	-
	S1_261533697	Phvul.006G101600	Redox ascorbate and glutathione glutathione GME	-	−1.30	-
FWS	S1_425572697	Phvul.010G025400	Disease resistance protein (TIR–NBS–LRR class) family	*-*	*-*	−4.39
	S1_425572697	Phvul.010G025500	Disease resistance protein (TIR–NBS–LRR class) family	*-*	*-*	−3.06
	S1_425572697	Phvul.010G026400	Disease resistance protein (TIR–NBS–LRR class) family	*-*	*-*	−2.12
	S1_425572697	Phvul.010G027500	Disease resistance protein (TIR–NBS–LRR class) family	*-*	*-*	−3.16
	S1_425572697	Phvul.010G027801	Disease resistance protein (TIR–NBS–LRR class) family	*-*	*-*	−4.78
	S1_425572697	Phvul.010G028700	Disease resistance protein (TIR–NBS–LRR class) family	*-*	*-*	−5.26
	S1_425572697	Phvul.010G028800	Disease resistance protein (TIR–NBS–LRR class) family	*-*	*-*	−2.59
	S1_425572697	Phvul.010G029000	Disease resistance protein (TIR–NBS–LRR class) family	*-*	*-*	−2.76
	S1_425572697	Phvul.010G029100	Disease resistance protein (TIR–NBS–LRR class) family	*-*	*-*	−4.11
	S1_425572697	Phvul.010G029700	Disease resistance protein (TIR–NBS–LRR class) family	*-*	*-*	−3.14

*4DPI* 4 days post-inoculation, *6DPI* 6 days post-inoculation, *24HPI* 24 h post-inoculation, *CG* count of galls, *CEM* count of egg masses, *FWS* Fusarium wilt severity

^*^The expression values refer to those reported by Santini et al. (2016) according to log2 fold-change (FC) ≥ 1.0 and false discovery rate ≤ 0.05 and **by Chen et al. (2019) according to log2 fold-change (FC) ≥ 2.0

Specifically for co-infection, the genes identified on chromosome Pv06 encoding redox ascorbate and glutathione (Phvul.006G094500) and pathogenesis-related (PR) proteins (Phvul.006G109000) were the only ones that overlapped with those differentially expressed in common bean after infection with Mi (log2FC = 1.07 and − 3,87; Santini et al. 2016) or Fop (log2FC = − 2.92 and − 3.39; Chen et al. 2019). This suggests the involvement of these genes in controlling defense responses against both pathogens.

The *loci* on chromosome Pv10 identified for both traits—gall count and egg mass count—under Mi + Fop co-infection include genes encoding putative disease resistance proteins previously described by Chen et al. (2019) as differentially expressed in common bean 24 h after inoculation with Fop. The cluster of ten TIR–NBS–LRR class genes on Pv10 is an important candidate region targeted by both pathogens, and it may play a crucial role as a key regulator in Mi and Fop co-infection in common bean (Table 6).

## Discussion

The most effective and sustainable strategy for controlling plant diseases is the development of resistant cultivars. To enable this development under a molecular breeding strategy, the first step is identification of genomic *loci* associated with partial or complete resistance responses. Through genome-wide association studies (GWAS), a targeted search for SNP markers linked to these important genomic *loci* can facilitate marker-assisted selection. Recent mapping efforts have revealed that disease resistance in common bean is controlled by multiple genomic regions, with distinct *loci* associated with specific resistance traits.

In this study**,** inferences could be made regarding the magnitude of genetic variability within the population studied, and as a diversity panel was used, these results were expected. Gall count showed moderate heritability estimates (Mi = 0.68 and Mi + Fop = 0.74), followed by egg mass count (Mi = 0.54 and Mi + Fop = 0.59). These results are consistent with those reported by Giordani et al. (2021), who found heritability estimates of 0.55 for the galling index and 0.49 for egg mass count for the same population used in this study. In a broader perspective, inheritance of these traits varies among populations and may be controlled by major dominant genes or by additive effects, depending on the genetic background (Pesqueira et al. 2025; Ferreira et al. 2012).

Heritability estimates for Fusarium wilt severity showed moderate to low values (Fop = 0.52 and Mi + Fop = 0.22). Paulino et al. (2021) reported similar results for Fop resistance as expressed in the disease severity rate (DSR) when studying a similar population and the same Fop isolate (h^2^ = 0.63). Resistance to Fusarium wilt in common bean has been associated with several genes that contribute small to moderate effects in resistance levels (Torres et al. 2022; Leitão et al. 2020). Specifically, when infection is preceded by nematode inoculation, the lower heritability estimate indicates even more complex control of the trait, suggesting that resistance to Fop alone does not explain the response of bean accessions to this disease complex.

Freitas et al. (2025) has evaluated resistance responses to Fop and *Meloidogyne incognita* in a F8 recombinant inbred line (RIL) population (n = 73), derived from crosses between the nematode-resistant cultivar ‘IAC-Tybatã’ and the susceptible cultivar ‘Branquinho’ (Giordani et al. 2021). The authors found six RILs (1, 5, 8, 25, 31, and 47) that exhibited low infection levels by both *Fusarium oxysporum* f. sp. *phaseoli* (Fop) and root-knot nematodes under co-infection.

Gall formation by RKN in plants reflects early infection success and plant cell reprogramming. In this context, resistance is often strongly influenced by plant recognition/defense genes, such as R genes, and signaling pathways (Abad and Williamson 2010; Kyndt et al. 2013). In contrast, egg mass formation involves not only infection, but also nematode development, fecundity, and plant tolerance, which are affected by host physiology, environmental conditions, and plant resource allocation. Therefore, this trait is more likely to be polygenic and environmentally sensitive, leading to lower heritability.

GWAS revealed eight genomic regions associated with plant responses to infection by the root-knot nematode *M. incognita*. Regions on chromosomes Pv02, Pv04, and Pv05 were associated with responses to egg mass count in plants inoculated solely with Mi, whereas in co-infection, regions on Pv10 and Pv11 were detected. Regarding gall count, regions associated with resistance to Mi alone were identified on Pv07, Pv08, and Pv10, whereas in co-infection with Fop, regions were identified on Pv05, Pv06, Pv10, and Pv11.

Giordani et al. (2021), in previous genome-wide association and QTL mapping studies using the same population, identified intervals on chromosomes Pv06, Pv07, Pv08, and Pv11 linked to egg mass production, while the gall index was mainly associated with *loci* on chromosomes Pv01, Pv02, Pv05, and Pv10. In our study, *loci* on chromosome Pv10 was consistently detected, highlighting this region as a robust genomic hotspot for Mi resistance. The other *loci* previously reported were not confirmed under our experimental conditions and analytical approach.

Floriani et al. (2025) also mapped QTLs on Pv10 using a segregating population derived from the cross between the genotypes ‘IAC-Tybatã’ and ‘Branquinho’, establishing that the genetic architecture of the root-galling index for nematode resistance is polygenic. The QTL on Pv10 covers a region of 102 genes, 23 of which were also studied by Orsi et al. (2025), highlighting the importance of *loci* on chromosome Pv10 in genetic control of nematode resistance. Together, these findings indicate a polygenic and trait-dependent architecture of resistance, with galling and egg mass phenotypes governed by partially distinct yet overlapping genomic regions.

Moreover, meta-analyses on QTLs for disease resistance in common bean have shown that subtelomeric regions, particularly on Pv07, act as hotspots for defense-related *loci* (Rahmanzadeh et al. 2022), suggesting convergence between nematode resistance and broader disease resistance regions. In our study, GWAS also revealed two regions on Pv07 associated with resistance to Fop based on disease severity, as well as one region on Pv08. This result is consistent with previous studies by Leitão et al. (2020) and Paulino et al. (2021) in which genes related to common bean defense against Fop were found on chromosome Pv07, reconfirming that it is an important region in the control of Fusarium wilt disease.

The main biological processes associated with all traits in this study included signal transduction, proteolysis, protein phosphorylation, and oxidation–reduction. Together with molecular binding functions, with the membrane as the main cellular component, these processes affect plant responses to biotic stimuli, such as nematode and Fop effectors, activating a tightly coordinated network of cellular responses (Shukla et al. 2018a, b).

Upon effector recognition by plasma membrane receptors, phosphorylation cascades involving MAPKs and calcium-dependent kinases rapidly activate transcription factors such as WRKY and ERF, leading to reprogramming of defense gene expression (Meng and Zhang 2013; Lu and Tsuda 2021; Shukla et al. 2018a, b; Nahar et al. 2011). Phosphorylation events also activate NADPH oxidases, resulting in a burst of reactive oxygen species (ROS), which function both as antimicrobial agents and as signaling molecules in defense-related pathways (Kimura et al. 2020). The modulation of this ROS burst depends on oxidation–reduction enzymes, including peroxidases, superoxide dismutases, and catalases, which maintain cellular homeostasis while reinforcing cell walls at attempted feeding sites. Recent studies have shown the importance of ROS modulation in resistance responses against Mi, Fop, and their co-infection (Chen et al. 2014; Ferreira et al. 2012; Giordani et al. 2021; Li et al. 2021; Luo et al. 2024; Pesqueira et al. 2025; Przybylska and Obrępalska-Stęplowska 2020; Warmerdam et al. 2020).

Finally, proteolysis, particularly through the ubiquitin–proteasome system, regulates the stability of key defense proteins by selectively degrading repressors of signaling pathways or modulating hormone signaling involving salicylic acid (SA), jasmonic acid (JA), and ethylene (ET). This modulation orchestrates the balance between localized resistance and systemic acquired resistance (SAR) (Marino et al. 2012; Verma et al. 2016; Thilakarathne et al. 2025). The main processes found to be associated with the studied traits strongly suggest that common bean responses to Mi and Fop involve an intricate interplay among signaling pathways, hormonal regulation, redox balance, and signal transduction pathways.

In the *loci* on Pv08 associated with gall count and on Pv07 associated with Fusarium wilt severity, we identified differentially expressed genes involved in calcium binding and signaling functions. Transcriptomic studies have shown that calcium signaling plays a critical role in plant immunity, mediating defense responses against pathogens by triggering characteristic Ca^2^⁺ spikes (signatures), leading to downstream ROS production, cell wall defenses, and defense gene activation (Lu and Tsuda 2021; Li et al. 2021; Bhar et al. 2023; Molinari et al. 2024). Particularly under co-infection, the simultaneous activation of these pathways generates a complex hormonal and oxidative environment, where ROS act as both defense signals and stress inducers (Luo et al. 2024).

Auxin-responsive factors were also found to overlap in plant responses against Fop and Mi, which were identified in *loci* on chromosomes Pv07 and Pv06, respectively. Specifically, the region on Pv06 associated with gall count under Mi and Fop co-infection suggests the important role of the gene Phvul.006G101300 in the Mi-Fop disease complex. In the early stages of infection, nematodes locally induce auxin biosynthesis, leading to giant cell expansion and lateral root formation (Gheysen and Mitchum 2019; Kyndt et al. 2016, 2013; Wang et al. 2018; Ren et al. 2024). When plants are co-infected with Fop, this early auxin accumulation creates a susceptible state that the fungus can subsequently exploit. At later infection stages, auxin interacts with other hormones, such as SA and JA, shaping the balance between growth and defense (Kidd et al. 2011; Xue et al. 2015). Thus, auxin can act as a temporal regulator of susceptibility and resistance, with its early activation favoring susceptibility, but its later downregulation leading to plant resistance.

On chromosome Pv02, genes related to secondary metabolism were identified as candidates associated with egg mass count. The coordinated induction of secondary metabolism also leads to robust oxidative and structural defenses, integrating phenolic compound accumulation and reinforcement of cell wall polymers to limit pathogen infection (Upadhyay et al. 2025; Yang et al. 2023). Enhanced lignin biosynthesis contributes to cell wall fortification, forming a physical barrier that restricts nematode migration and feeding site expansion. Likewise, flavonoid pathway genes play important roles in signal transduction and redox homeostasis, acting against nematode and Fop infections (Shukla et al. 2018a, b; Chen et al. 2019).

In analyzing the co-infection assay, it is noteworthy that two ascorbate–glutathione (AsA-GSH) related genes were identified on Pv06 and Phvul.006G109000 was reported to be differentially expressed under both Fop and Mi infections. The AsA–GSH pathway constitutes a critical redox regulatory module in plant defense, maintaining cellular redox homeostasis, supporting ROS signaling for defense activation, and limiting oxidative damage during biotic stress (Labudda 2018). Molinari et al. (2024) showed that early Mi infection in tomato suppresses immune responses but strongly activates the plant antioxidant system. Similarly, Mandal et al. (2008) reported that infection of tomato by *Fusarium oxysporum* activates antioxidant enzymes, including glutathione- and ascorbate-related peroxidases. These findings highlight the crucial role of antioxidants in the redox regulatory system in the early stages of plant defense against *M. incognita* and *F. oxysporum*. In this case, Phvul.006G109000 may be an important gene regulating the Mi–Fop disease complex.

Furthermore, on Pv06, the gene Phvul.006G109000, with functional annotation for a pathogenesis-related (PR) protein, was found to be differentially expressed under both Mi and Fop infections. This suggests it may play a role in bean response to co-infection. The PR proteins are a group that include enzymes such as chitinases and glucanases, which degrade pathogen cell walls, and proteins that strengthen plant cell walls and promote lignification (Ebrahim et al. 2011). Plants usually have low constitutive levels of both these enzymes, but they increase in response to biotic stress. Oliveira et al. (2012) showed that infection of cowpea roots by Mi led to increased chitinase and β−1,3-glucanase activity in a highly resistant cultivar. A few studies have also shown that the antimicrobial activity of PR proteins limits colonization of host plants by *F. oxysporum* (Narasimhan et al. 2003; Saikia et al. 2004). However, little is known about how these defense responses converge in co-infection.

Interestingly, responses to Fop infection were mainly associated with plant resistance (R) proteins of the NB-ARC–LRR family. Three genes of the NB-ARC and LRR family were found to be differentially expressed under Fop infection within a region on chromosome Pv07, identified under sole Fop inoculation. Under co-infection, a cluster with ten genes of the TIR class was identified on Pv10. These R genes are known to detect pathogen effectors and activate immune signaling. The NB-ARC domain controls activation through conserved motifs, while the LRR domain mediates effector recognition. Depending on their N-terminal region, TIR or CC domains trigger downstream defense responses and hypersensitive cell death (Dangl and Jones 2001; Zhou and Zhang 2020).

Proteins of the NB-ARC–LRR family are constitutively and ubiquitously expressed in plants, but the level of expression is typically low. This constitutive expression supports the hypothesis that R genes encode surveillance proteins that directly or indirectly detect effector molecules from pathogens and initiate an effective defense response (Williamson and Kumar 2006). Expression profiling and transcriptome studies have shown upregulation of these genes in resistance reactions in several crops, suggesting their involvement in early immune signaling and defense activation against a broad spectrum of pathogens, including Mi and Fop (Santini et al. 2016; Wen et al. 2017; Chen et al. 2019; Goyal et al. 2020; Warmerdam et al. 2020; Alekcevetch et al. 2021). Thus, this gene cluster represents promising candidates for resistance in genotypes under co-infection and warrants additional validation.

The genomic regions and markers associated with Mi and Fop resistance represent important elements for advancing our understanding of pathogen-host relationships within this complex pathosystem. Information on defense responses leading to resistance to Mi and Fop, both alone and in co-infection, is limited. Here, our hypothesis involved a subset of the genetic responses potentially involved in resistance. Our findings on genomic regions associated with common bean responses to Mi, Fop, and their co-infection, along with evidence of genes differentially expressed during infection by the respective pathogens alone, can provide new insights and directions in breeding this crop.

Improving our understanding of resistance mechanisms and identifying new R genes is important for implementing marker-assisted selection to develop cultivars with long-lasting resistance to the disease complex studied. Functional validation of the genes identified as potentially associated with resistance is the next step and should be carried out in future studies to elucidate the molecular mechanisms involved in this disease complex. Confirming the role of these resistance genes will be a major step forward for common bean breeding, as it will enable introgression of this resistance into elite bean cultivars.

## Data Availability

The data are available upon request from the corresponding author.

## Abbreviations

RKN: Root-knot nematode
Fop: *Fusarium oxysporum* F. sp. *phaseoli*
Mi: *Meloidogyne incognita*
SNP: Single-nucleotide polymorphism
GBS: Genotyping by sequencing
SDW: Sterile distilled water
QTL: Quantitative trait loci
DSR: Disease severity rate
ROS: Reactive oxygen species
JA: Jasmonic acid
ET: Ethylene
SAR: Systemic acquired resistance
ERF: Ethylene-responsive factor
MAPK: Mitogen-activated protein kinase
PR: Pathogenesis related
